# Potential effects of lipid lowering and antioxidant activity of exosomes from Phascolosoma esculenta: *in vivo* and *in vitro* studies

**DOI:** 10.3389/fphar.2025.1560462

**Published:** 2025-03-25

**Authors:** Lin Yang, Xue Yi, Meitao Duan, Ahmed Mahal, Binbin Chen, Zhiqiang Zhang, Jungang Ren, Ming Chen, Chen Meng, Moxun Xu, Ahmad J. Obaidullah, Chen Wang, Mingyuan Liu

**Affiliations:** ^1^ College of Pharmacy, Jiamusi University, Jiamusi, China; ^2^ Key Laboratory of Functional and Clinical Translational Medicine, Xiamen Medical College, Fujian Province University, Xiamen, China; ^3^ Department of Medical Biochemical Analysis, College of Health Technology, Cihan University-Erbil, Erbil, Iraq; ^4^ Department of Pharmacy, Xiamen Xianyue Hospital, Xiamen, Fujian, China; ^5^ Department of Pharmaceutical Chemistry, College of Pharmacy, King Saud University, Riyadh, Saudi Arabia

**Keywords:** phascolosoma esculenta, lipid lowering, exosomes, hypolipidemic, antioxidant

## Abstract

Exosomes released from cells have been shown to play an important role in health and disease due to their potential application in therapy and diagnosis. First of all, we extracted the exosome from Phascolosoma esculenta, and studied the lipid lowering and antioxidant activity of Phascolosoma esculenta exosome. It was found that Phascolosoma esculenta exosomes could significantly decrease the lipid accumulation caused by oleic acid, and improve triglycerides (TG), total cholesterol (TC), high-density lipoprotein cholesterol (HDL-C) and low density lipoprotein (LDL-C). The exosomes of Phascolosoma esculenta also exhibit antioxidant activity, which can decrease the amount of reactive oxygen species (ROS) and malondialdehyde (MDA), and raise the level of superoxide dismutase (SOD) and total antioxidant capacity (T-AOC). The exosomes of Phascolosoma esculenta have the function of reducing lipid and antioxidation.

## 1 Introduction

Hyperlipidemia (HLP) is a chronic metabolic disease characterized by an increase in the circulating levels of lipids or lipoproteins (Liu et al., 2023). It mainly consists of TC, TG, LDL, and HDL, all of which may be excessively expressed in the blood. The level of LDL is positively correlated with atherosclerosis, heart disease, and coronary artery indices, making it a crucial factor in cardiovascular diseases [(Joint Committee on revision, 2017; Zokaei et al., 2020; Zeljković et al., 2019; Zhao, 2019; Zhang et al., 2018)]. Dyslipidemia can stimulate ROS production, weaken antioxidant defenses, increase lipid peroxidation, and lead to redox imbalance, oxidative stress, and metabolic alterations (Yue et al., 2023). Long-term oxidative stress further consumes fatty acids in the livers of individuals with hyperlipidemia, promoting fatty liver degeneration and resulting in tissue cell damage, injury, and even death (Jiang et al., 2024). Recent studies have shown that there is a close relationship between oxidative stress and pathophysiology in cardiovascular diseases (Yan et al., 2018). Oxidative stress is considered a plausible mechanism underlying hyperlipidemia. Antioxidants are increasingly recognized as potential hypolipidemic drugs (Wu et al., 2019).

Phascolosoma esculenta is a marine invertebrate endemic to China, also known as Haiding and bamboo coral. It is produced in China’s coastal areas, such as Fujian, and Taiwan Province (Wu, 2020). Phascolosoma esculenta is recognized as ‘marine cordyceps sinensis’ and can be used as food and medication. Research suggests that Phascolosoma esculenta exhibits the effects of relaxing smooth muscle, lowering blood pressure as well as antithrombotic, antioxidant, antibacterial, antiviral, anti-aging, anti-inflammatory, and anti-tumor properties [(Peng, 2022; Zhou et al., 2006; Zhou et al., 2007; Jiang et al., 2004; Shen et al., 2003)].

Exosomes are a type of extracellular vesicles that possess a lipid bilayer membrane with a saucer-like shape, and the diameters generally range from 30 nm to 200 nm (Johnstone et al., 1987). Almost all cells can secrete exosomes, which are widely distributed in bodily fluids such as plasma and urine and have the function of cellular messengers, participating in cellular communication (Banliat et al., 2020). Exosomes contact target cells via floor molecules and switch the contents to the cytoplasm of target cells, thereby altering the physiological state of recipient cells (Colombo et al., 2014; Thery et al., 2002). It naturally has corrected target-homing specificity and can move the organic barrier *in vivo* (Skryabin et al., 2020). Due to these characteristics, exosomes play a widespread role in treatment. In addition, various proteins in exosomes, such as antioxidant enzymes and anti-inflammatory factors, have been demonstrated to regulate and enhance the management of hyperlipidemia, to protect vascular endothelial cells, and to decrease the formation of atherosclerotic plaques [(Kobayashi et al., 2018; Li et al., 2020; Palazzolo et al., 2020; Gardiner et al., 2016)]. However, there is no study on the extraction of exosomes from Phascolosoma esculenta. The effect and mechanisms of its anti-hyperlipidemia are uncertain and need further study.

In this paper, the exosomes of Phascolosoma esculenta was treated as the research object, using differential and ultracentrifugation strategies to extract exosomes [(Cvjetkovic et al., 2014; Konoshenko et al., 2018; Zeringer et al., 2015)]. Using TEM and NTA techniques to identified the morphology and structure of exosomes, the lipid-lowering effect and antioxidant activity of Phascolosoma esculenta exosomes *in vitro* was also measured, which furnished a foundation for subsequent hypolipidemic mechanism research (Herrera-Balandrano et al., 2021; Ferramosca et al., 2019). This study provides a new approach to promote the prevention and treatment of hyperlipidemia, and offers new ideas for discovering anti-hyperlipidemia drugs.

In addition, we also studied mice fed a high-fat diet *in vivo*. It provides additional insights for understanding the anti-hyperlipidemia effect of Phascolosoma esculenta exosomes. This study will further understand the role of Phascolosoma esculenta exosomes in promoting health, and lay a foundation for the future development of food rich in this kind of function.

## 2 Materials and methods

### 2.1 Materials and chemicals

Phascolosoma esculenta was bought from Fuzhou Minji Sea Worm Jelly Co. Ltd. (China, Fuzhou). Procurement of HepG2 cells fromXiamen Medical College. Oxidative stress index MDA, T-AOC, and SOD detection kit, Lipid index TG, TC, HDL-C and LDL-C detection kits were purchased from Solarbio Technology Co, Ltd. (China, Beijing). All other analytical chemicals are from Yuanye Biotechnology Co, Ltd. (China, Shanghai).

### 2.2 Extraction and identification of exosomes of Phascolosoma esculenta

#### 2.2.1 Extraction

A working solution was prepared by mixing 0.25% trypsin digestive enzyme and type IV collagenase in a 1:1 volume ratio. The body wall tissue was cut, placed in the working solution, and digested for 20 min. An equal volume of DMEM medium was added to the working solution to terminate the digestion process. The mixture was then centrifuged for 10 min at 300 g, then centrifuged for an additional 15 min at 2,000 g, and finally for 30 min at 10,000 g. After the last centrifuging step, the sediment is removed and the supernatant is kept. The supernatants were collected by passing through a filter membrane of 0.22 µm diameter. Transfer the filtrate into an EP tube and centrifuged for 20 min at 2,700 rpm at 4°C to enrich the exosomes. The enriched solution was then centrifuged for 70 min at 120 000 g at 4°C. After this last centrifuging step, the precipitate is the desired exosome.

#### 2.2.2 Identification

##### 2.2.2.1 Transmission electron microscopic identification of exosomes (TEM)

Take the exosome sample from −80°C, dissolve it on ice, and gently blow it with a pipette to make the sample thoroughly mixed. 20 μL of exosome suspension was dropped into the copper mesh of the electron microscope and keep the suspension on the copper mesh for 5 min. Using 2% uranyl acetate solution for negative dyeing and fixation for 5 min, absorbing excess liquid with filter paper, and naturally drying at room temperature. Finally, the copper mesh with the fixed sample was put under a 120 kv transmission electron microscope to observe and take photos.

##### 2.2.2.2 Nanoparticle tracking analyzer (NTA)

Take 20 µL of exosome suspension and dilute it to 1,000 µL with deionized water. Use a 1 mL syringe to load the sample. Set the cycle time and operate according to the instructions of the NS300 NTA. Start the test on the computer and record the results.

##### 2.2.2.3 Lipidomic analysis of exosomes

A lipid sample extracted from Phascolosoma esculenta was submitted to theXiamen Life Internet Technology Co., Ltd. for analysis. Data for lipid molecular specie were presented as mol% of the total lipids analyzed.

### 2.3 Hypolipidemic and antioxidant activities of exosomes of Phascolosoma esculenta *in vitro*

#### 2.3.1 Cell culture

After mixing HepG2 cells with cell culture medium (DMEM medium containing 10% FBS and 1% double antibiotic), add them to a Petri dish. Incubate the culture dish in a cell incubator with 37°C and 5% CO_2_. Change the medium every 48 h, and conduct passage when the cell growth density reaches 80%–90%.

#### 2.3.2 *In Vitro* cytotoxicity activity

The CCK-8 approach was used to detect the toxicity of Phascolosoma esculenta exosomes to HepG2 cells. HepG2 cells (5 × 10^3^ cells/plate) were seeded into a 96-well plate and incubated for 24 h. Then, the supernatant was removed, add concentrations of exosomes (0, 10, 50, 100, 200, 300, 400, 500 μg/mL) of Phascolosoma esculenta, and tradition for 24 h beneath the above conditions. To the plate was added 4 μL of serum free medium containing 10% CCK8. After incubation for 2 h, the absorbance at 450 nm was measured with an enzyme labelled apparatus. Through the calculation of the survival ratio of HepG2 cells, the optimum concentration of Phascolosoma esculenta was obtained.

#### 2.3.3 Induction high-fat HepG2 cells

The excessive accumulation of lipids in HepG2 cells was induced by oleic acid as a model fat source. HepG2 cells (2 mL, 1 × 10^6^ cells) were inoculated on a 6-well culture plate and incubated for 24 h. Cell adhesion changes the culture medium and induces excessive lipid accumulation in HepG2 cells with 0.2 mmol/L oleic acid. The exosomes with different concentrations were added and cultured for 24 h.

#### 2.3.4 Oil red O staining

After incubation, HepG2 cells were subjected to OROS staining kit, and the results were as follows: Observed under an inverted microscope.

#### 2.3.5 Detection of lipid indexes

The induction and treatment methods of high-fat HepG2 cells are described in Section 2.3.3. Then, the cells were collected and lysed on ice bath. Lipid indexes (TC, HDL-C, TG, and LDL-C) were detected according to detection kits method.

#### 2.3.6 Detection of oxidative stress indexes

According to the ROS kit instructions, cells have been inoculated into a 6-well plate. After establish a model and administer medication, 200 µL/well of DCFH-DA (diluted 1:1,000) was added and further diluted in a serum-free medium. The cells were then cultured at 37°C for 20 min. Afterward, the cells were washed with serum-free medium three times and images were captured under a fluorescence microscope. Each group had three replicate wells, and for each well, three non-overlapping fields were selected for imaging. The ImageJ software was used to quantitatively analyze the ROS levels. Additionally, we detected SOD, MDA, T-AOC, and other biomarkers in the cells following the manufacturer’s instructions.

### 2.4 Hypolipidemic and antioxidant activities of exosomes of Phascolosoma esculenta *in vivo*

#### 2.4.1 Animal hyperlipidemia model

C57BL/6J mice were obtained from Basic Medical College of Xiamen Medical College (n=72). The mice were adaptively fed in a standard cage for one week, with the ambient humidity of 60% and the temperature of 25°C ± 1°C, and provided with standard diet and water. Mice were randomly divided into 64 mice and 8 normal controls. In the present study, the hyperlipemia animal model was established with a high fat diet for 6 weeks. The serum lipid index (TC, TG) of hyperlipemia model group was significantly higher than that of normal controls (P < 0.01) which met the modeling conditions. The normal control group (n = 8) during the follow-up intervention. HFD was administered to the model (n = 8), 6 treatment groups (n = 8), and simvastatin control. Based on the experimental results, the dosage of Phascolosoma esculenta exosome was 750 μg/ml, 1,000 μg/ml, 1,500 μg/ml, 2,000 μg/ml, and 2,500 μg/ml respectively. Simvastatin positive drug group was given simvastatin 5 mg/kg by gavage. To eliminate the effect of the method of administration on the experiment, the control group (PBS group) received PBS by gavage. At the end of the intervention, eye blood samples were collected Serum was obtained by centrifugation at 4°C (3,000 rpm/10 min), and the lipid and oxidative. The stress index of serum was analysed. Liver samples were taken, and the liver weight was measured with normal saline solution. A portion of the liver was used for HE staining and oil red O staining, and the remainder of the liver was used for the analysis of lipid and oxidative stress. The experimental design was approved by Xiamen Medical College’s Experimental Animal Ethics Committee.

#### 2.4.2 Imaging of small animals *in vivo*

The distribution of exosomes in mice was studied by *in vivo* imaging technology of small animals, and its changing law *in vivo* and *in vitro* was explored, so as to provide basis for further clarifying its mechanism of action. Exosomes labeling uses lipophilic dye Dio to dye exosomes.

#### 2.4.3 Drug toxicity experiment

Serum biochemical indexes (AST and ALT levels) were analyzed with appropriate commercial kits (Beijing Solaibao Technology Co., Ltd.).

#### 2.4.4 Blood lipids determination of the serum

Serum lipid indexes (including TG, TC, HDL-C and LDL-C levels) were analyzed with an appropriate commercial kit (Beijing Suolaibao Technology Co., Ltd.).

#### 2.4.5 Oxidative stress indicator determination of the serum

Oxidative stress indicators, including SOD, T-AOC activities, as well as MDA content in the serum, were analyzed using appropriate kits (Solarbio Science and Technology Co., Ltd., Beijing).

#### 2.4.6 HE and oil red o staining of liver tissue

The mouse liver was soaked in 4% neutral paraformaldehyde, washed with distilled water, and then placed in 70% ethanol overnight, Paraffin embedding, 4 μm slicing, sealing and HE staining. Liver tissue was embedded in OCT and frozen for 8 mm, and stained with oil red. The pathological changes of liver in each group were observed under 200 times optical microscope.

### 2.5 Data analysis

Data were analyzed by means of a one-way analysis of variance or unpaired *t*-tests to determine Group differences (Graph Pad Prism 9.0). Results were considered When P < 0.05, statistical significance was observed. All data in this paper are means ± standard Deviation of more than or equal to three separate experiments.

## 3 Results

### 3.1 Identification of Phascolosoma esculenta exosomes

**FIGURE 1:**
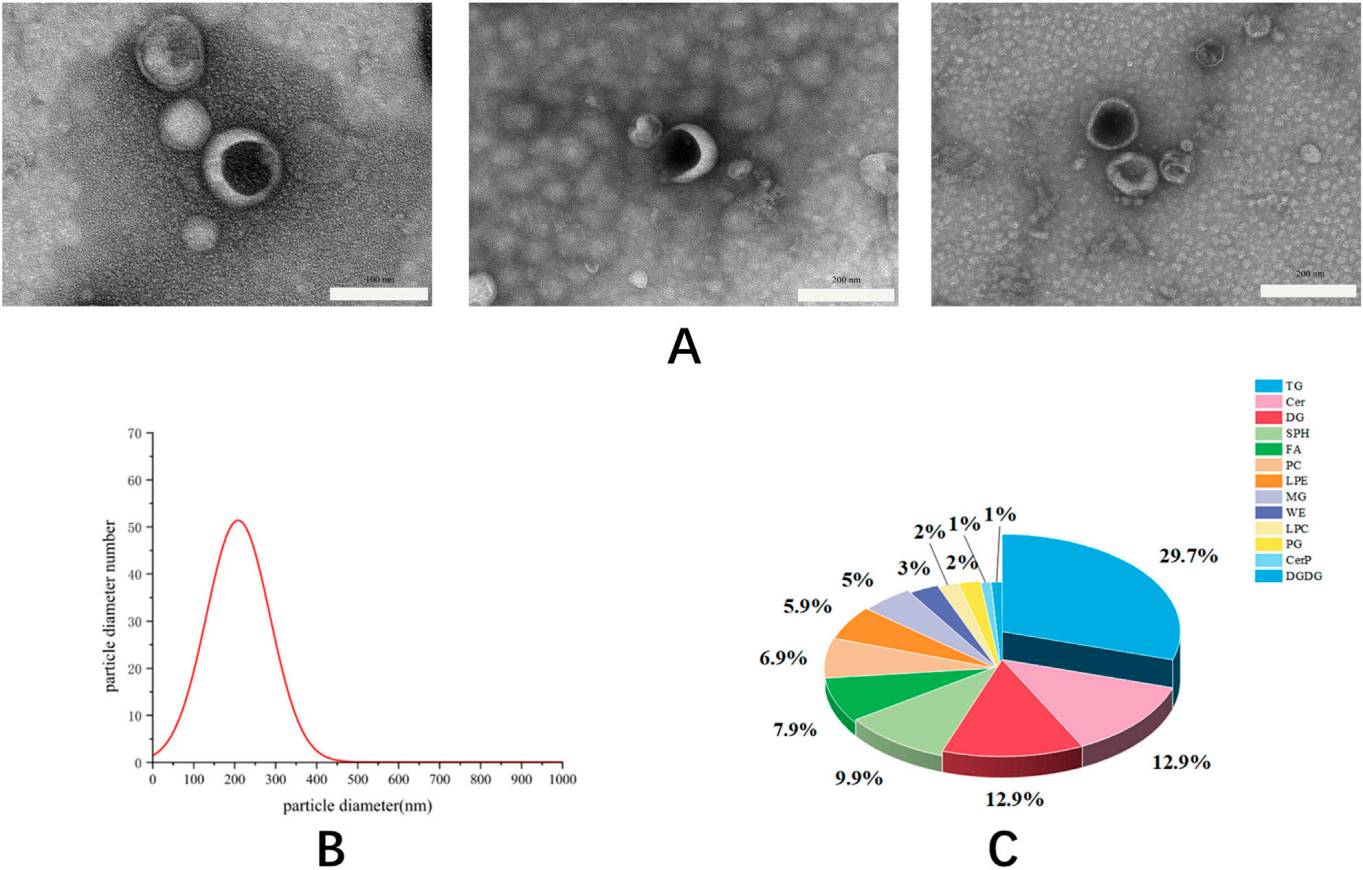
The image of exosomes taken by transmission electron microscope with a scale **(A)**. The particle size distribution of exosomes was measured by nanoparticle tracking analysis **(B)**. The types of lipids contained in exosomes determined by ultra-high performance liquid chromatography-mass spectrometry **(C)**.

In this study, differential centrifugation was used to separate the exosomes of Phascolosoma esculenta, Further characterization of exosomes such TEM, NTA and Lipidomic Analysis was also performed. Result showed that the exosomes of Phascolosoma esculenta can be separated and enriched through differential centrifugation, and the giant particles, fibers, and insoluble components in the uncooked substances can be eliminated simultaneously. The image acquired with the aid of TEM confirmed that the exosomes of Phascolosoma esculenta were nearly saucer in shape and evenly disbursed (Figure 1A). The common particle dimension of the exosomes of Phascolosoma esculenta measured by means of NTA was in the range of 100–200 nm (Figure 1B). The types of lipids contained in exosomes determined by ultra-high performance liquid chromatography-mass spectrometry and the result showed that exosomes contained TG (∼29.7% of total lipids), Ceramide (Cer, ∼12.9% of total lipids), DG (∼12.9% of total lipids). (Figure 1C). The morphology, particle dimension and lipid components met the definition preferred of exosomes.

### 3.2 Hypolipidemic effect of Phascolosoma esculenta exosomes in cells

#### 3.2.1 Cytotoxicity of Phascolosoma esculenta exosomes

**FIGURE 2:**
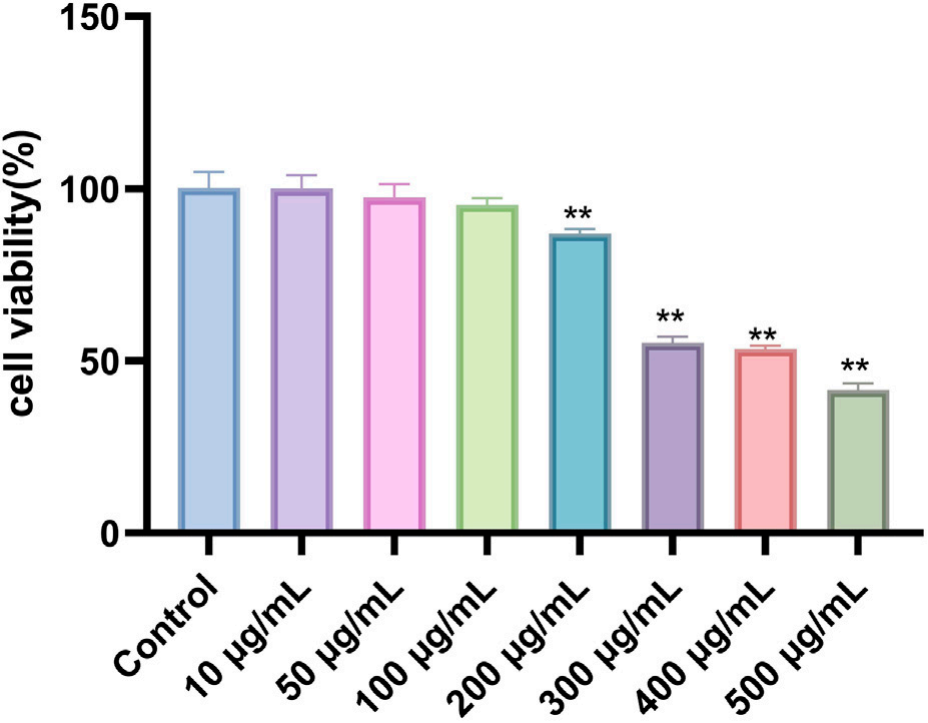
Evaluation of HepG2 cells survival rate incubated with Phascolosoma esculenta exosomes for 24 h. Note: Compared with the control group, *p < 0.05, **p < 0.01.

We assessed the cytotoxicity of Phascolosoma esculenta exosomes and the viability of HepG2 cells using the CCK-8 assay. HepG2 cells were treated for 24 h with Phascolosoma esculenta exosomes (0, 10, 50, 100, 200, 300, 400, 500 μg/mL). The results demonstrated a dose-dependent influence of Phascolosoma esculenta exosomes on the survival rate of HepG2 cells. The cell survival rate was above 90% at concentrations below 100 μg/mL, suggesting that the concentration was no cytotoxicity (Figure 2). Consequently, the concentration of Phascolosoma esculenta exosomes was determined to be 0–100 μg/mL in further studies.

#### 3.2.2 Effect of Phascolosoma esculenta exosomes on lipid accumulation

We characterized intracellular lipid accumulation using lipid index and OROS to confirm the successful establishment of the high-fat HepG2 cell model. Compared the model group with the control group, we found that although HDL-C contents dramatically dropped, TC, TG, and LDL-C contents greatly increased. Furthermore, the increase of intracellular lipid droplets was also noted by OROS. The high-fat model was successfully established in HepG2 cells, as these data verified. Then, to ascertain the oil concentration that will provide the greatest reduction of fat accumulation, a preliminary screening investigation was conducted to evaluate the impact of Phascolosoma esculenta exosome concentration 0–100 μg/mL on HepG2 cells stimulated by oleic acid. According to OROS data, the amount and intensity of lipid droplets in HepG2 cells treated with Phascolosoma esculenta exosomes were reduced in comparison with the model group. Compared with the model group, the results showed that the HDL-C in the treatment group increased and the TG and TC levels decreased. The lipid-lowering impact becomes more pronounced with an increase in exosome concentration, indicating that the effectiveness of Phascolosoma esculent exosomes on hyperlipidemia is dose-dependent. The Phascolosoma esculenta exosomes-treated cells exhibited a considerable reduction in the quantity of lipid droplets and a lighter color intensity compared to the model group, as determined by the OROS method. These findings demonstrated that in high-fat HepG2 cells, the accumulation of lipid droplets was decreased by the Phascolosoma esculenta exosomes.

**FIGURE 3:**
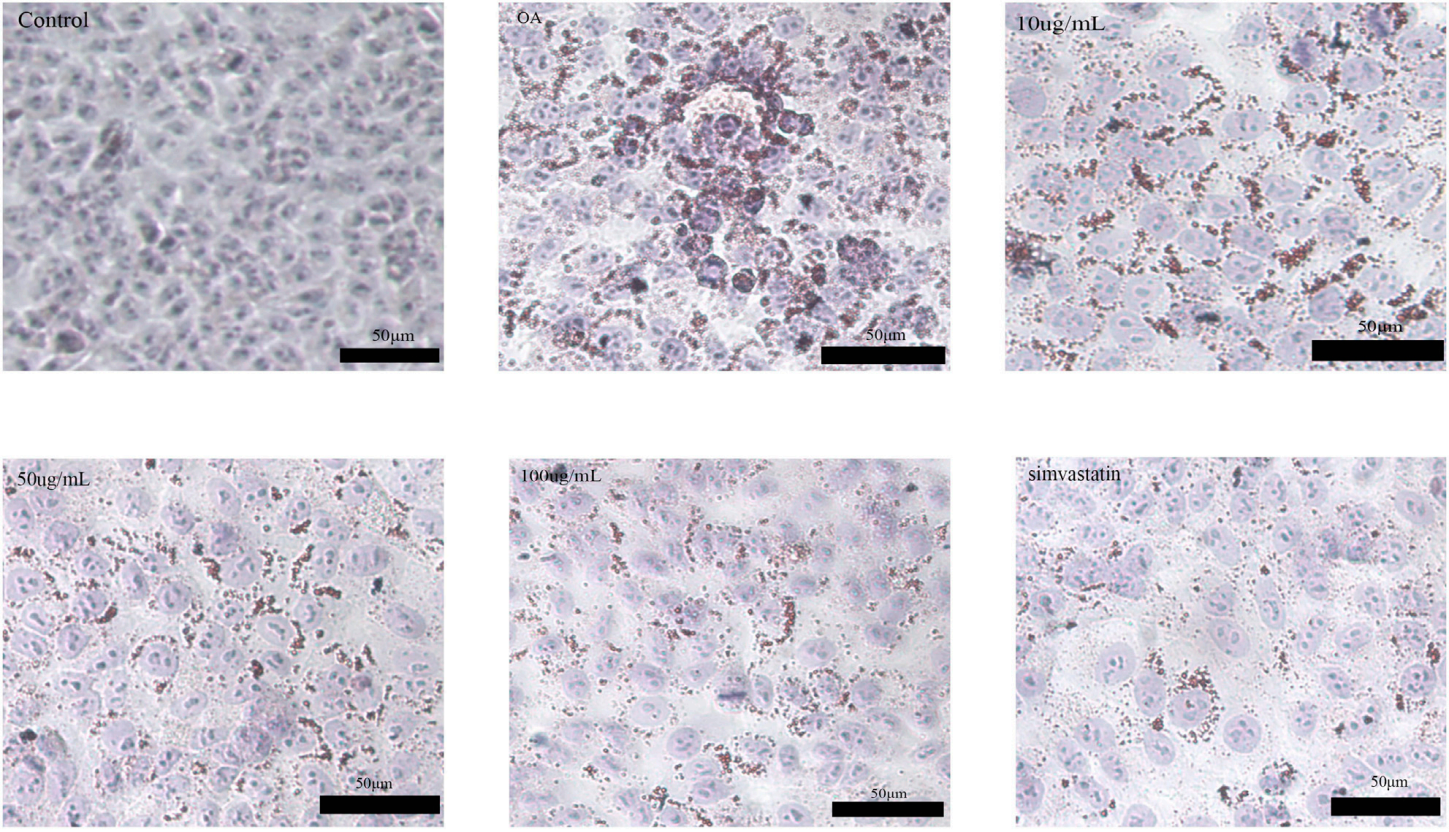
Oil red O staining image in oleic acid-induced high-fat HepG2 cells.

**FIGURE 4:**
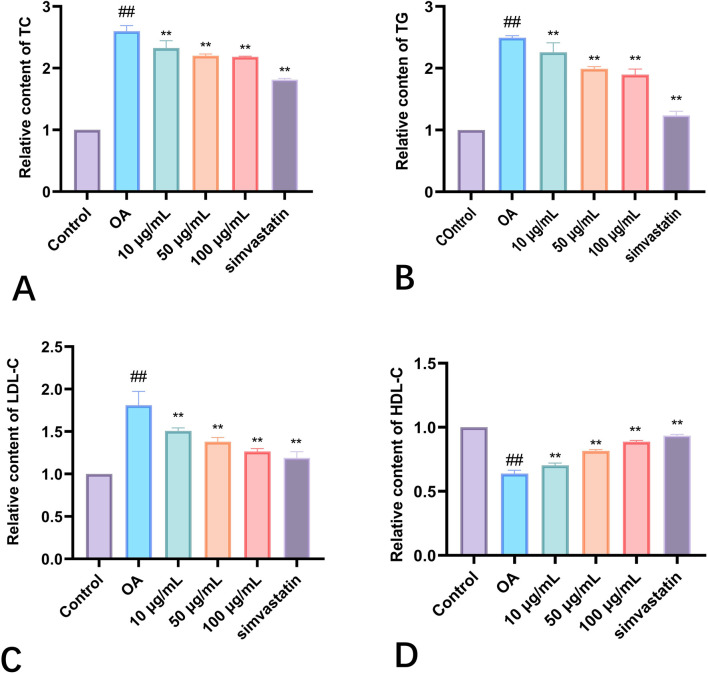
Effects of different concentrations of exosomes of Phascolosoma esculenta on TC **(A)** TG **(B)** LDL-C **(C)** HDL-C **(D)**. Note: compared with the control group, ^#^P < 0.05, ^##^P < 0.01. Compared with the OA group, *P < 0.05, **P < 0.01.

An imbalance in the metabolism of triglycerides and cholesterol is the primary sign of hyperlipidemia (Figure 3). Consequently, an analysis was conducted on the TC and TG levels in HepG2 cells. The group treated with Phascolosoma esculenta exosomes had significantly lower TG and TC levels (*P* < 0.01, respectively) in comparison to the model group (Figures 4A, B). This demonstrates the ability of these two drugs to prevent hyperlipidemia. Furthermore, high-fat HepG2 cells' LDL-C content was lowered by Phascolosoma esculenta exosome treatment (Figure 4C). It can lessen the symptoms of hyperlipidemia by raising the HDL-C level (Figure 4D).

### 3.3 Effects of Phascolosoma esculenta exosomes on oxidative stress *in vitro*

**FIGURE 5:**
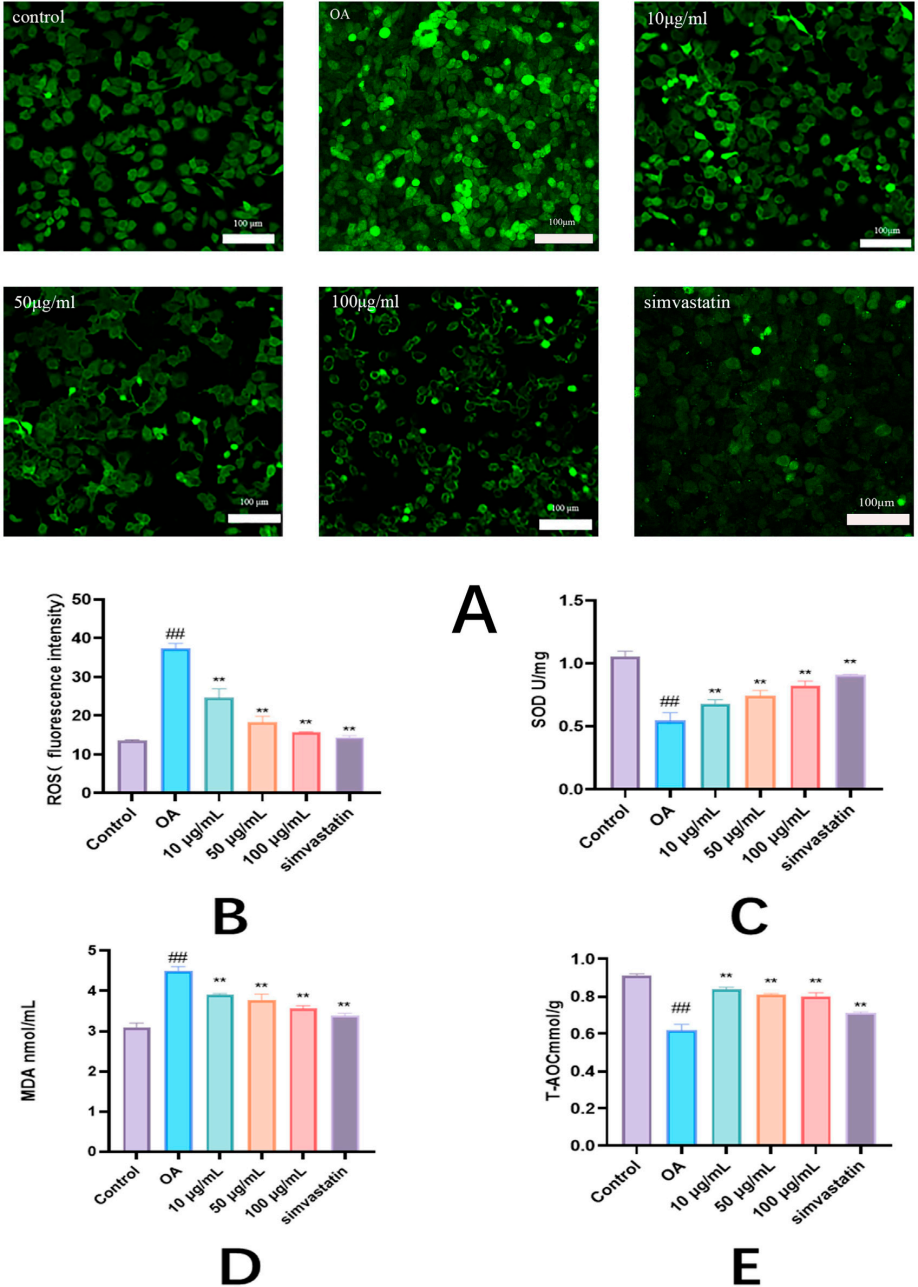
Antioxidant effects of exosomes of Phascolosoma esculenta on the levels of reactive oxygen species (ROS) **(A)** ROS fluorescence intensity **(B)** superoxide dismutase (SOD) **(C)** malondialdehyde (MDA) **(D)** and total antioxidant capacity (T-AOC) **(E)** in high-fat HepG2 cells induced by oleic acid. Note: compared with the control group, ^#^P < 0.05, ^##^P < 0.01. Compared with the OA group, *P < 0.05, **P < 0.01.

ROS was significantly reduced by the exosomes than in the model cells (*P* < 0.01) (Figure 5B). Superoxide dismutase (SOD) activity was measured to avoid oxidative stress caused by free radicals in cells. It was found that the SOD activity in model cells was obviously decreased (*P* < 0.01). Compared with the control group, the SOD activity was obviously higher in the treatment group than in the model group. It shows how both of these compounds enhance the antioxidant potential of cells (Figure 5C). Since MDA is a secondary reaction product produced in the last step of lipid peroxidation, the evaluation of MDA concentration may provide additional information on cellular oxidation status. Higher levels of MDA lead to more free radicals that can destroy vital metabolism and physiology. As shown in the graph, lipid peroxidation was observed in the model cells, which indicated that the MDA content was significantly higher (*P* < 0.01). Compared to model cells, MDA levels were lower after treatment with Phascolosoma esculenta (Figure 5D). High levels of fat in the blood will also decrease T-AOC’s total antioxidant ability, so that the body cannot effectively eliminate oxygen free radicals. As can be seen in the diagram, the T-AOC in the model group was reduced, suggesting that HepG2 was under oxidative stress. On the other hand, the level of T-AOC in the exosomes was higher than that in the model group (*P* < 0.01) (Figure 5E). The results showed that PHC could reduce the lipid peroxidation of HepG2 cells with high fat content. Thus, Phascolosoma esculenta exosome has the potential to regulate the antioxidative enzyme system, increase the antioxidative effect, and reduce the oxidative stress induced by oleic acid in high fat HepG2 cells.

### 3.4 Hypolipidemic effect of phascolosoma esculenta exosomes *in vivo*

#### 3.4.1 Imaging of small animals *in vivo*

**FIGURE 6:**
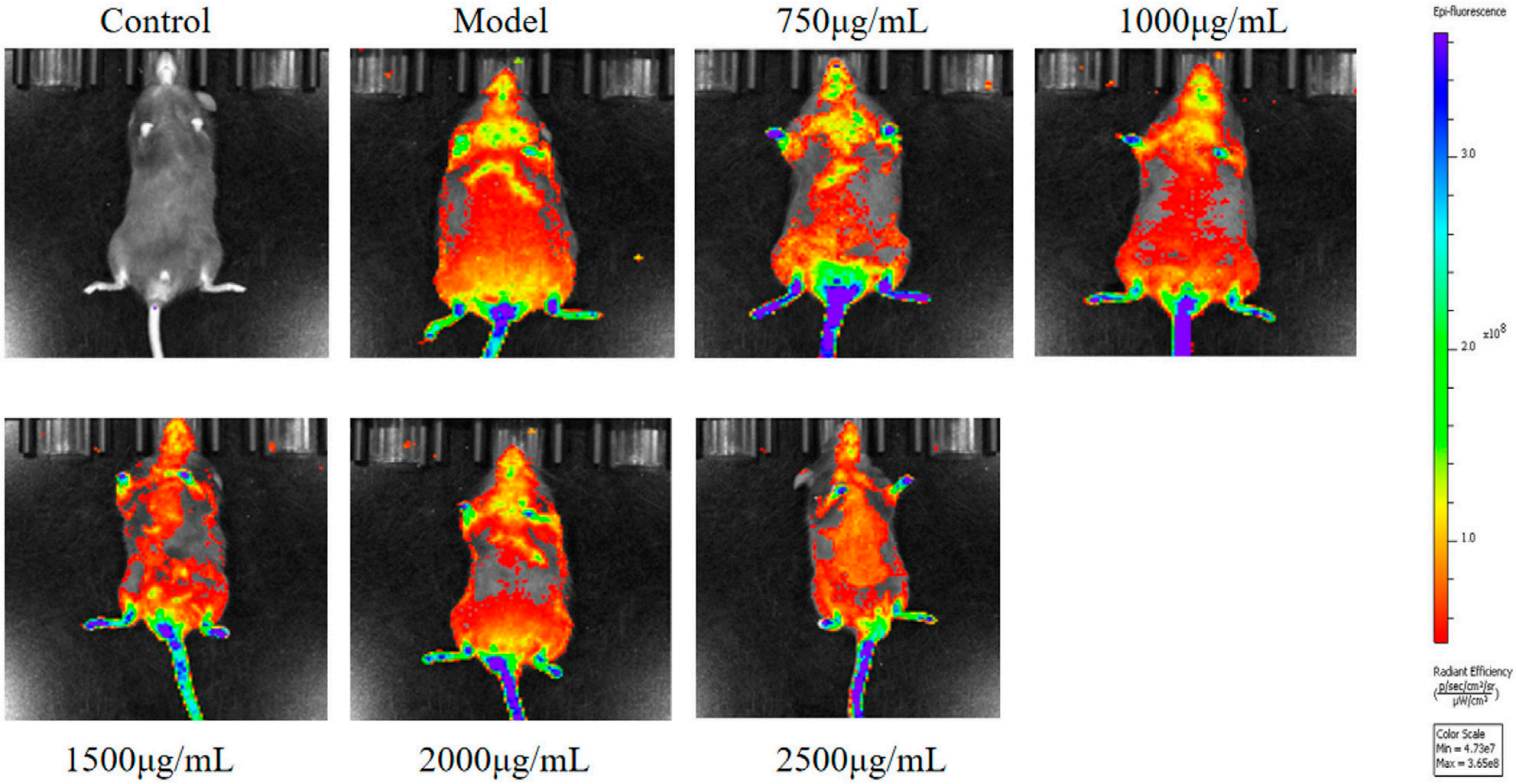
Representative IVIS images of mice injected with Dio labeled Exo. IVIS imaging was performed 24 h after injection.

In order to analyze the distribution of exosomes in the body, we tracked the exosomes labeled by Dio. *In vivo* imaging system (IVIS) showed that exosomes labeled with Dio were mainly sent to liver, liver, spleen and lung (Figure 6).

#### 3.4.2 Effect of Phascolosoma esculenta exosomes on drug toxicity in mice

**FIGURE 7:**
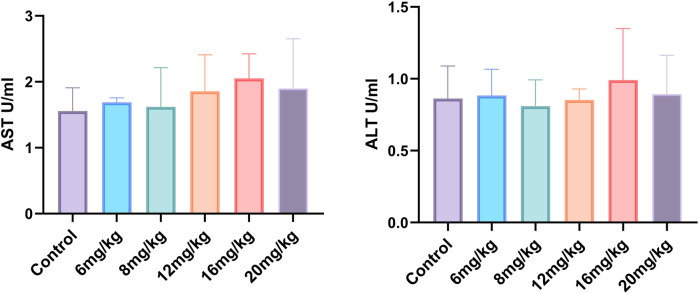
Effect of Phascolosoma esculenta exosomes on drug toxicity in mice.

After the high-fat mouse model was established by feeding high-fat feed, the Phascolosoma esculenta exosomes were injected into the tail vein for administration. After 4 weeks of continuous administration, blood samples were taken to detect AST and ALT indexes in liver. Figure 7 shows that there is no statistical difference between the administration group and the blank group. It is preliminarily concluded that the selected concentration gradient of Phascolosoma esculenta exosomes has no toxic effect on mice.

#### 3.4.3 Effect of Phascolosoma esculenta exosomes on lipid accumulation in serum and liver of mice

**FIGURE 8:**
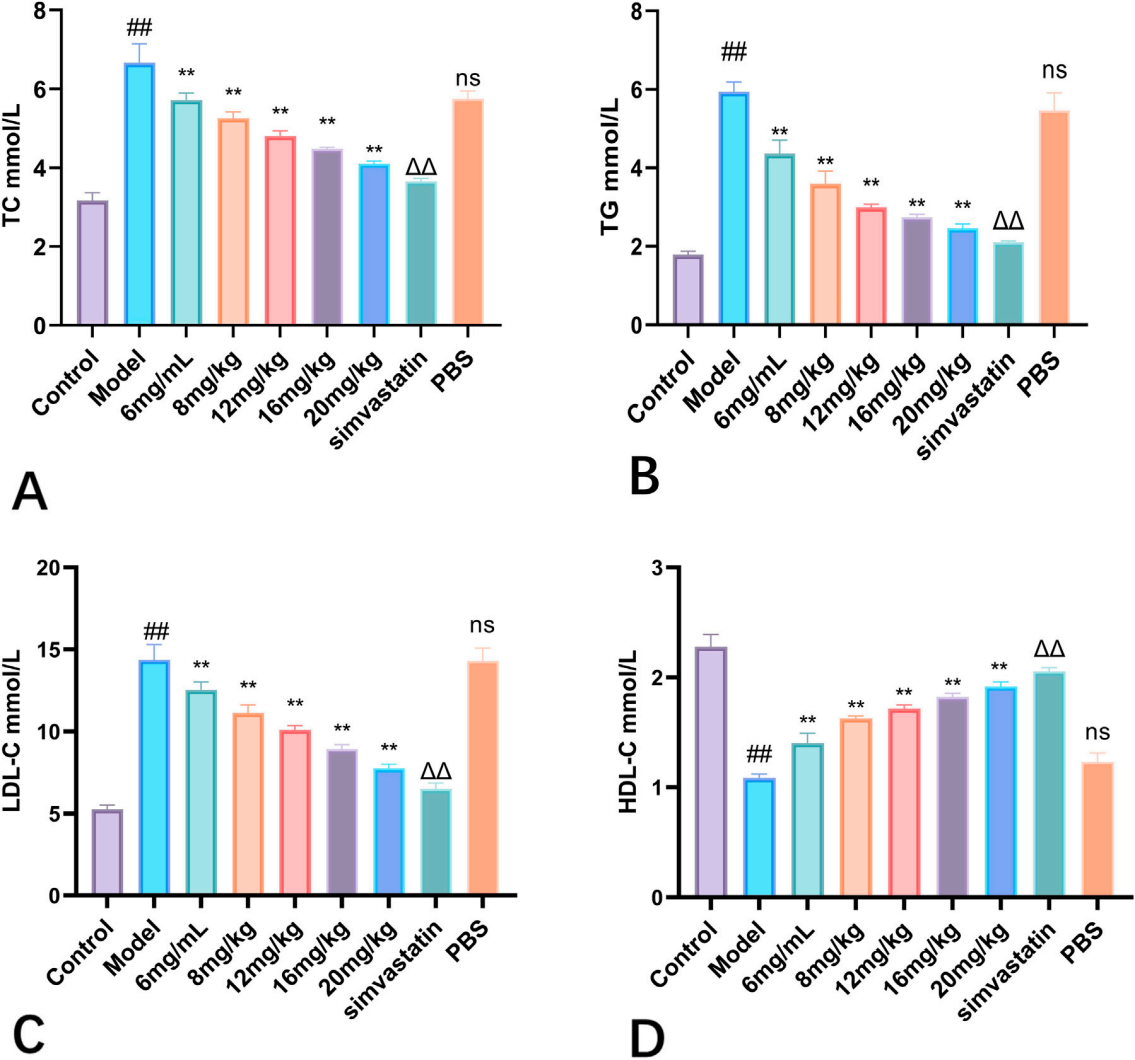
Effects of different concentrations of exosomes of Phascolosoma esculentaon TC **(A)** TG **(B)** LDL-C **(C)** HDL-C **(D)**. Note: Compared with the control group, ^#^P < 0.05, ^##^P < 0.01, Compared with the model group, ns P > 0.05, *P < 0.05, **P < 0.01. Compared with PBS group, ΔP < 0.05, ΔΔ P < 0.01.

In this study, the effects of Phascolosoma esculenta exosomes on lipid metabolism were studied in mice. To study the preparation of high-fat animal model by feeding high-fat diet with high-fat feed. On this basis, its therapeutic effect on hyperlipidemia mice was discussed. The results showed that TC, TG and LDL-C in the model group were higher than those in the control group (*P* < 0.01), but HDL-C was significantly reduced (*P* < 0.01), which showed that the rat model of hyperlipemia was established successfully. Moreover, high fat diet can also influence the lipid metabolism in mice. After four weeks of treatment with different doses of Phascolosoma esculenta exosomes, the mice were euthanized for serum analysis. The results indicated that TC, TG, LDL-C and HDL-C were significantly lower than those in model group (Figure 8). Furthermore, the effect of the high dose group on the exosome of PHASCOLOSOMA esculenta was lower than that of simvastatin group, but it was significantly different from the control group. It is suggested that Phascolosoma esculenta exosomes can improve the lipid and glucose metabolism of high fat diet.

#### 3.4.4 Effect of Phascolosoma esculenta exosomes on serum oxidative stress in mice

**FIGURE 9:**
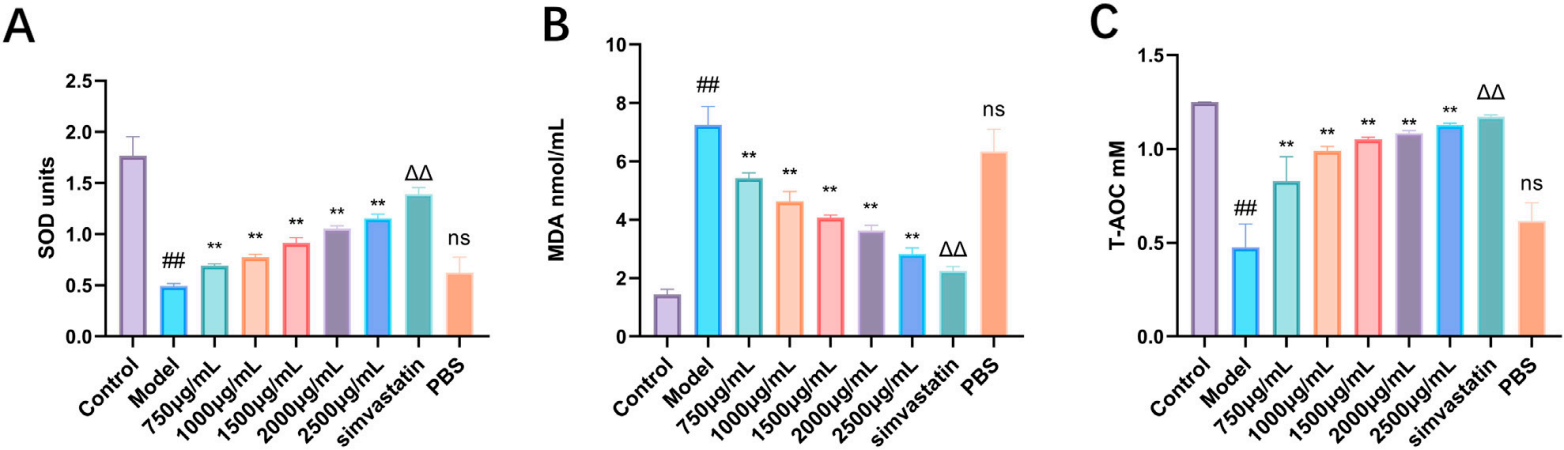
Antioxidant effects of exosomes of Phascolosoma esculenta on the levels of superoxide dismutase (SOD) **(A)** malondialdehyde (MDA) **(B)** and total antioxidant capacity (T-AOC) **(C)** in hyperlipidemia mouse model. Note: Compared with the control group, ^#^P < 0.05, ^##^P < 0.01. Compared with the model group, ns P > 0.05, *P < 0.05, **P < 0.01. Compared with PBS group, ΔP < 0.05, ΔΔP < 0.01.

Hyperlipidemia will lead to dysfunction of multiple organs of the body and trigger oxidative stress. Therefore, this study analyzed the indexes of oxidative stress in serum. The activities of SOD and T-AOC in serum of mice in high-fat diet group decreased significantly, and the level of MDA increased significantly, which may be related to high-fat diet (Figure 9). Different doses of Phascolosoma esculenta exosomes can significantly increase the levels of SOD and T-AOC in serum, and it is dose-dependent. Similarly, compared with the model group, the MDA level in the Phascolosoma esculenta exosomes treated with different doses decreased gradually. Therefore, we speculate that the Phascolosoma esculenta exosomes can play a role in reducing blood lipid by reducing the oxidative stress of the body.

#### 3.4.5 HE and oil red o staining of liver tissue

**FIGURE 10:**
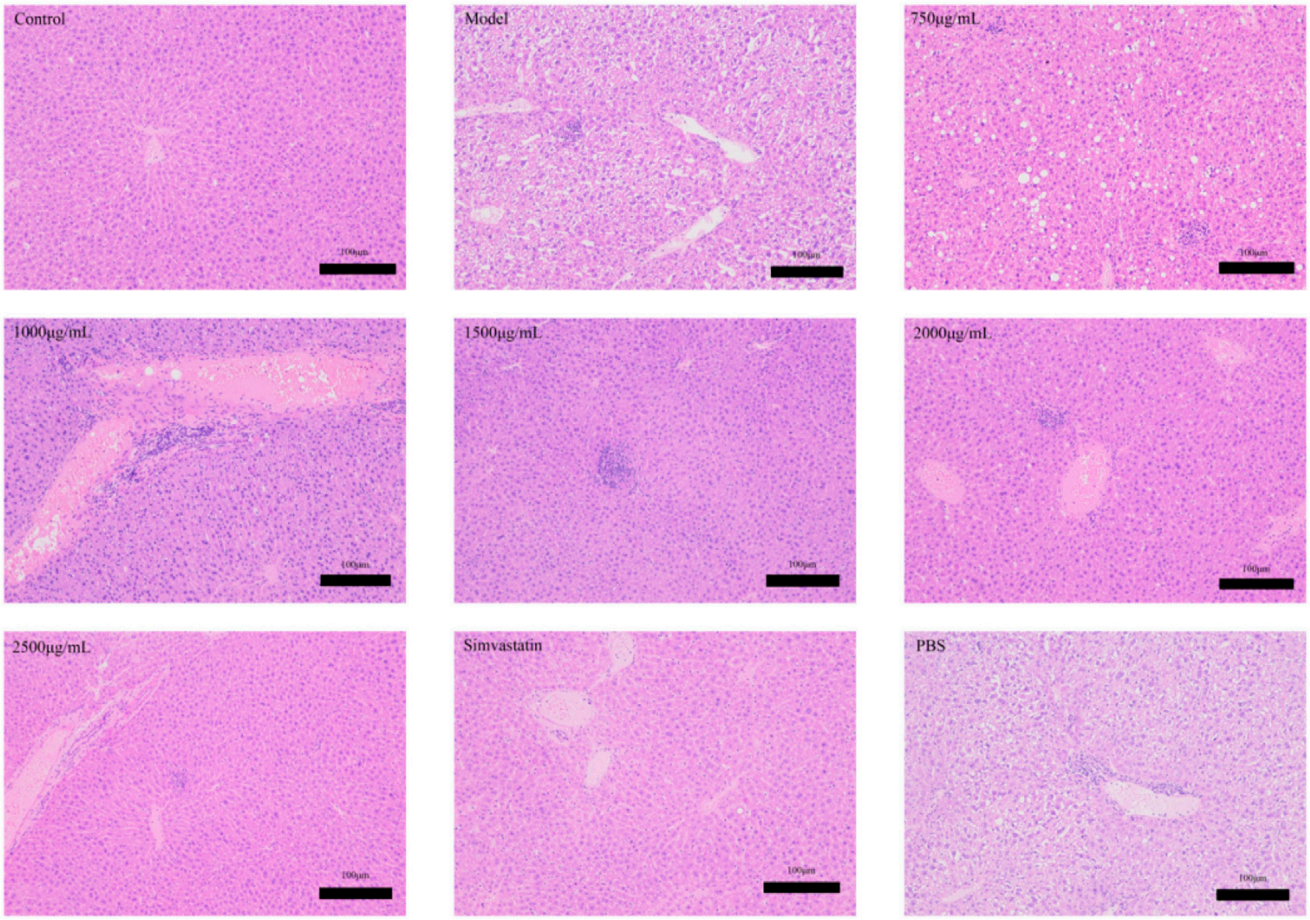
Effects of Phascolosoma esculenta exosomes and simvastatin on liver H&E staining in induced high-fat mice.

**FIGURE 11:**
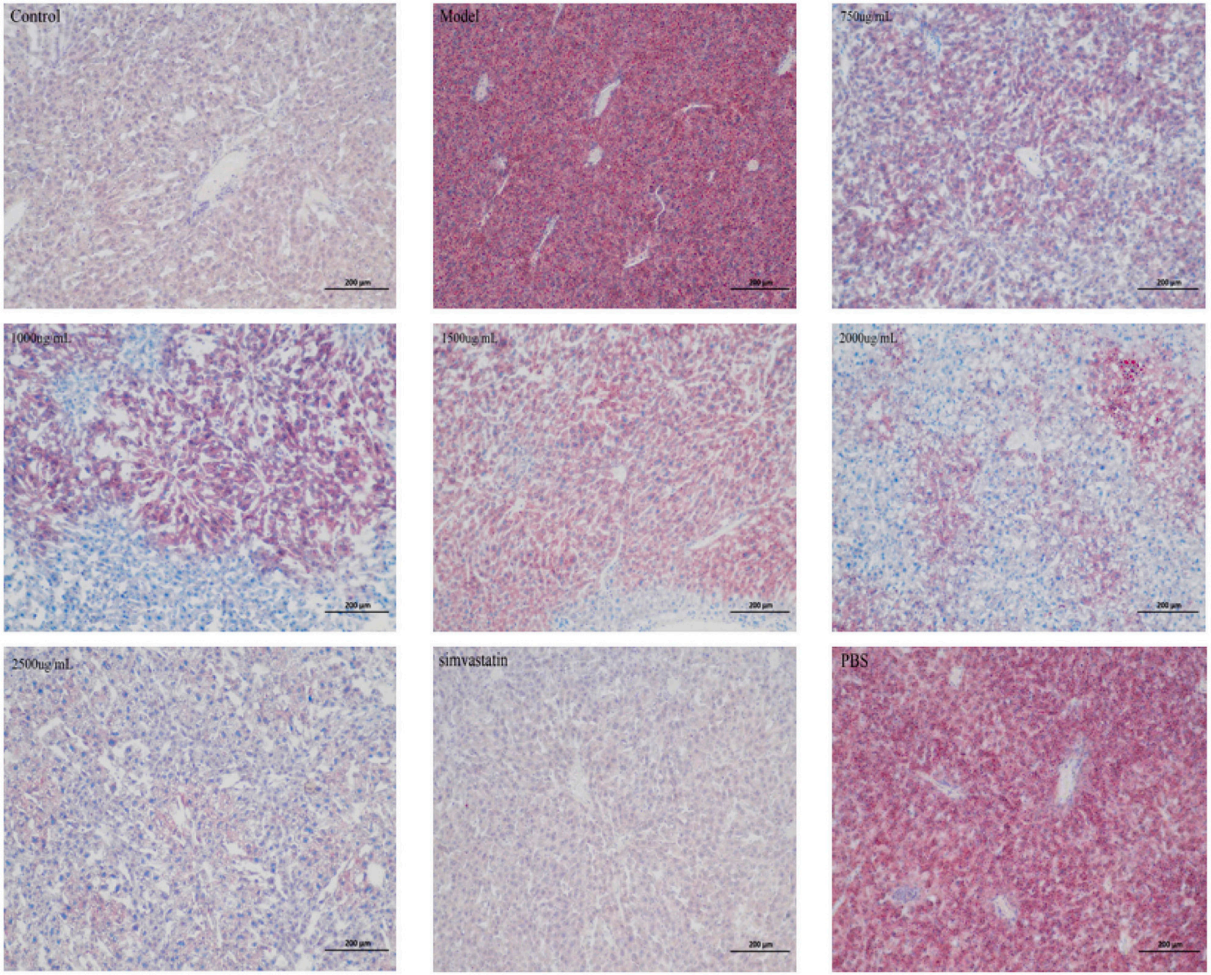
Representative images of Oil Red O staining of the liver samples from indicated groups.

Dyslipidemia is a common disease in patients with hyperlipidemia. In this experiment, the cell structure of the liver in the control group is normal, as shown in Figure 10. Conversely, the liver tissue of hyperlipemia model mice showed cytoplasm vacuole and steatosis In the model group, there was a significant increase in the area of fat injury compared with the control group. Along with the increasing of the intervention dosage, the lipid lesions decreased gradually, and eventually disappeared. The liver, on the other hand, tends to maintain a normal structure. In Figure 11, we found that the lipid was reduced and the lipid droplets distributed uniformly. The results showed that there was no significant accumulation of red fat drops in the liver of the control group, but the liver fat drops in the model group were significantly increased, and the majority of them were flake. Compared with the model group, the liver morphology of exosomes and treated with simvastatin was significantly decrease in lipid deposition in liver.

## 4 Discussion

This study used differential centrifugation and ultracentrifugation to extract Phascolosoma esculenta exosomes for the first time. *In vitro* experiments have been performed to investigate the hypolipidemic impact of Phascolosoma esculenta exosomes. The results received from *in vivro* and *vivo* experiments exhibit that the lipid-lowering impact of Phascolosoma esculenta exosomes is associated to oxidative stress relief. In addition, our *in vitro* findings also established the remarkable lipid-lowering effect of Phascolosoma esculenta exosomes. It was additionally found that treatment with Phascolosoma esculenta exosomes can alleviate oxidative stress in a dose-dependent manner. Further hypolipidemic mechanism study is ongoing.

Phascolosoma esculenta is a dual-purpose resource for both medication and food, rich in nutrients and possessing genuine pharmacological activity. It has the potential to be developed into practical foods and even medicines. If successfully developed into lipid-lowering health foods or medicines, it could have significant financial and social value, particularly in the prevention and treatment of atherosclerotic cardiovascular and cerebrovascular diseases. This would mark an impressive transformation from being a “Fujian-famous food” to a “world-renowned medicine”.

## Data Availability

The raw data supporting the conclusions of this article will be made available by the authors, without undue reservation.
